# Impact of Social Determinants of Health in the Care of Moebius Syndrome: A Case Report

**DOI:** 10.7759/cureus.45297

**Published:** 2023-09-15

**Authors:** Brooke Schwartz, Jessie Limonta, Malka Goodman, Katherine Semidey

**Affiliations:** 1 Pediatrics, Florida International University, Herbert Wertheim College of Medicine, Miami, USA; 2 Pediatrics, Nicklaus Children's Hospital, Miami, USA; 3 Pediatrics, United Heritage Institute CommunityCare Clinic, Miami, USA

**Keywords:** moebius sequence, children with special needs, uninsured patients, interdisciplinary care, social determinants of health

## Abstract

Moebius syndrome is a rare congenital disorder characterized by nonprogressive uni- or bilateral abducens nerve (VI) and facial nerve (VII) palsy. Other cranial nerves (CN) such as CN III, IV, and IX-XII may be involved leading to varied presentations. Speech development, communication, and psychomotor complications are common. Given the complexity of the disease, patients require an individualized, multidisciplinary care plan involving many medical specialists. Accessing this level of care without insurance poses seemingly insurmountable challenges and places immense financial strain on both patients with Moebius syndrome and their families. Physicians must advocate for patients with Moebius syndrome and be knowledgeable about the community resources available to them such as non-profit organizations. This report presents a case of Moebius syndrome in an uninsured, immigrant, 13-year-old male and the barriers to providing him with adequate care.

## Introduction

Moebius syndrome is a clinically diagnosed, rare congenital disorder characterized by non-progressive uni- or bilateral abducens nerve (VI) and facial nerve (VII) palsy. Prevalence is estimated to be 1/250,000 live births [1]. Involvement of other cranial nerves (III, IV, and IX-XII) may also occur leading to a variety of craniofacial, odontological, orthopedic, and ophthalmological presentations [2].

Moebius syndrome patients can present with facial paralysis, poor vision, limited extraocular eye movements, concomitant autism spectrum disorder, behavioral disturbances, limb abnormalities, sensorineural hearing loss, dental caries, difficulty swallowing, and unintelligible speech. Given the complexity of Moebius syndrome presentations, management largely relies on supportive care by a multidisciplinary team [3]. Among this team are ophthalmologists, optometrists, neurologists, surgeons, psychiatrists, psychologists, physical therapists, occupational therapists, dentists, audiologists, and special educators. The cost of obtaining this kind of specialty care is not possible for all Moebius syndrome patients. Social determinants of health like low socioeconomic status and access to health insurance play a major role in our patient case. Here, we present a case of an uninsured, low-income, immigrant boy with Moebius syndrome and the healthcare management challenges we faced in treating him.

## Case presentation

Consent was obtained from the patient and family for the following case presentation.

An overweight recently immigrated, a 13-year-old male with previously diagnosed Moebius syndrome presented to our free health clinic for a well-child visit and management of his syndrome. The patient was born vaginally in Colombia at 38 weeks gestation. Pregnancy was complicated by polyhydramnios and birth was complicated by the need for forceps assistance. He remained hospitalized for the first month of life due to hypotonia and feedings requiring a nasogastric tube. Past surgical history was significant for correction of clubfoot at one year of age, tonsillectomy at seven years of age, and eyelid correction at nine years of age.

The patient immigrated to the United States (US) one month prior with his older brother, mother, and mother’s boyfriend through a Mexico-Arizona border crossing. They boarded a total of six flights from Colombia to Miami, FL, and were then held in an immigration detention center for three days before being released. The patient’s mother and her boyfriend were unemployed and looking for work. They lived with the patient’s uncle, whom they relied on for transportation, housing, and financial support. Additionally, the family only spoke Spanish and experienced a major language barrier upon arriving in the US.

The patient’s mother decided to take the family on the long and difficult journey to the US because they did not have access to the medical specialists needed to manage Moebius syndrome in Colombia. In Colombia, the patient’s only care was in a special needs classroom where some therapy was provided and lesson plans were tailored to his cognitive level. Unfortunately, the family’s strained financial situation here in the US had been a major barrier to obtaining specialized care for him given the patient's lack of eligibility for free (Medicaid) or low-cost (Florida KidCare) insurance. Without insurance, out-of-pocket costs for therapies and medical specialists were far too expensive for the family to afford. In addition, without an individualized education plan (IEP) for school, his mother had concerns that the patient would be starting middle school in a regular classroom, lacking any of the special attention and care he needed.

Upon initial evaluation, the patient struggled with verbal communication due to his diminished jaw mobility and sensorineural hearing loss. He relied heavily on hearing aids, however, during their journey to the US, his left hearing aid fell out and broke. Prior to the initial visit, he had been using only one hearing aid for the past month, which had already caused notable regression in his verbal communication. He was nearly nonverbal and communicated mainly through gestures. In Colombia, his medication regimen was working well and included clonidine and quetiapine for agitation and sleep disturbance. However, these were confiscated from him at the border. Without his medications, his mother was administering NyQuil, which helped with his nocturnal panic attacks, but his overall behavior and agitation had worsened. Additionally, he was using over-the-counter eye drops for eye irritation.

On physical exam at the time of initial presentation, the patient appeared younger than his stated age. He demonstrated bilateral facial palsy, limited jaw opening, lack of horizontal extraocular movements, and limited vertical extraocular movement. A surgical scar was present along the lateral canthus of the left eye. The patient also demonstrated difficulty dorsiflexing his feet. Additionally, he had very limited and poorly understood speech. Other findings included signs of cognitive delays, such as not following simple tasks from family members and behaving in a childlike manner. Due to his cognitive delay and unintelligible speech, a vision screen was unable to be assessed.

With limited finances and referral choices, our free clinic pediatric care team had to rely on nonprofit organizations and the public school system for management. We began by providing the patient with a prescription for psychoeducational evaluation, the first step in developing an IEP for placement in special education classes. This prescription was provided to his public school to prompt an evaluation at no cost to the patient. We also contacted The Advocacy Network on Disabilities, a local advocacy organization for children with special needs, to obtain disability aid resources. They recommended The Florida Elks Children’s Therapy Services, a local therapy program with grant funding for low-income families, where the patient eventually began receiving one hour of free speech therapy per week. They also assisted him in navigating the public school system’s Exceptional Student Education (ESE) courses.

The patient also acutely needed audiology care to obtain a new hearing aid. It was imperative for the patient to obtain access to a hearing aid to regain his recent verbal losses, understand the new linguistic world around him, and interact with his family and caregivers. We submitted an application to the Sertoma Speech and Hearing Foundation, a philanthropic arm of a Floridian audiology group, in hopes of obtaining a hearing aid at a lower cost. They conducted a comprehensive audiology exam, however, they recommended further specialist evaluation. An optometry appointment at our free clinic was made to evaluate his vision, however, due to the patient being nonverbal, an accurate exam was unable to be successfully completed. He was referred for a more highly specialized visual evaluation by ophthalmology. The patient also required a dental evaluation due to his challenges with oral hygiene due to limited jaw mobility. He was added to the list for our next free Dental Day with a local volunteer dentist. The patient's mother was provided with new prescriptions for clonidine and quetiapine, which the family was able to afford with the use of a GoodRx coupon. While there is a national Moebius Syndrome Foundation that offers additional resources such as transportation, it is only available to US residents.

Upon a recent follow-up with the patient's mother, we learned he has since been approved for Simply Medicaid health insurance. He is now able to see specialists in speech-language pathology, plastic surgery, otolaryngology, psychology, neurology, dentistry, and ophthalmology at the local children's hospital. He has yet to receive new hearing aids but is pending an appointment for a specialized sedated audiology evaluation. He gets behavioral therapy for five hours a day during the week and two hours each of physical and speech therapy on the weekends. He is now in eighth grade and has an IEP to be placed in special education classes. His behavior and sleeping are also much improved on his same medication regimen of clonidine and quetiapine. He no longer requires the NyQuil at night, as his panic attacks have resolved. He is seen regularly by a neurologist for adjustment of his medications. He has seen a maxillofacial surgeon and pediatric dentist to assist with his diminished jaw mobility and dental care. Additionally, he does specialized exercises to target oral musculature to improve his bite strength.

The patient no longer qualifies for follow-up at our free clinic due to his Medicaid coverage, however, he is receiving full, comprehensive care with subspecialty follow-up at the local children’s hospital. His mother says he has shown marked improvement both behaviorally and physically. A major takeaway from this patient’s case is the huge juxtaposition seen between his care before and after being approved for Simply Medicaid health insurance. As opposed to relying on nonprofit organizations and the public school system, the patient now has access to specialty care in a large hospital system and all of the therapies he requires.

## Discussion

The patient’s case presented our team with a multitude of challenges given the complexity of his presentation and his lack of Medicaid or Florida KidCare eligibility. Given his need for an interdisciplinary care team, and the family’s inability to afford out-of-pocket costs for specialists and therapies, we struggled to find him even a fraction of the services and care he would ideally receive. Our team had to rely on local nonprofit organizations and our local, overstressed public school system to provide our patients with what the US healthcare system couldn’t.

As a whole, Moebius syndrome patients most commonly present with a loss of facial expression and restricted lateral eye movements [4]. Involvement of the vestibulocochlear (VIII), glossopharyngeal (IX), and hypoglossal (XII) nerves can present with difficulty hearing, eating, and swallowing. There may also be drooling and indistinct speech [1]. Other clinical features include limb abnormalities such as syndactyly, brachydactyly, or absent digits and talipes. Additionally, patients may present with anomalies in the musculoskeletal system. These include brachial muscle defects, hypoplasia or absence of the pectoralis muscle, absence of the sternal head of the pectoralis major, Klippel-Feil anomaly, and scoliosis [5]. Oral-maxillofacial malformations associated with this syndrome include cleft lip and palate, underdevelopment of the maxilla, and ear deformities [5]. Patients may have early global developmental delay, however, they usually achieve all milestones by five years of age. While some studies report cognitive deficits being seen in only 10% of cases [1], others have varied widely ranging from 0 to 75% [6]. Some studies suggest patients with Moebius syndrome are more likely to have autism spectrum disorder with approximately 30-40% of children having some degree of autism [6]. However, the incidence of intellectual disability and autism spectrum disorders is questionable because diagnosis in these patients is challenging due to the effect of cranial nerve palsies on facial expression and eye contact [6].

In the neonatal period, the main focus of Moebius syndrome treatment is to restore normal breathing and feeding function [2]. Patients with difficulty swallowing or with tongue movements may require feeding tubes in infancy. Physical and speech therapy are needed to improve motor skills, coordination, and better control of speaking and eating abilities. With early speech therapy intervention, similar patients have great improvements in their speech [7]. Regular dental appointments are also necessary as these patients have difficulty removing food from their teeth and buccal spaces due to impaired jaw mobility, which can lead to poor dental health. Psychosocially, lack of facial expression can hinder attachments and affect social relationships, making psychologists an important component of the multidisciplinary team [8]. Ophthalmologic complications may also occur such as irritation, corneal dryness, and even corneal erosion due to the inability to blink and lack of tear production from the lacrimal duct [1]. Early surgical interventions also play a role in treatment. Gracilis muscle-free transfers for the development of smile expression (“smile surgery”) and procedures to correct strabismus and limb and jaw deformities have been reported [7,3]. As previously discussed, the care of this wide array of clinical anomalies requires numerous specialists.

**Figure 1:**
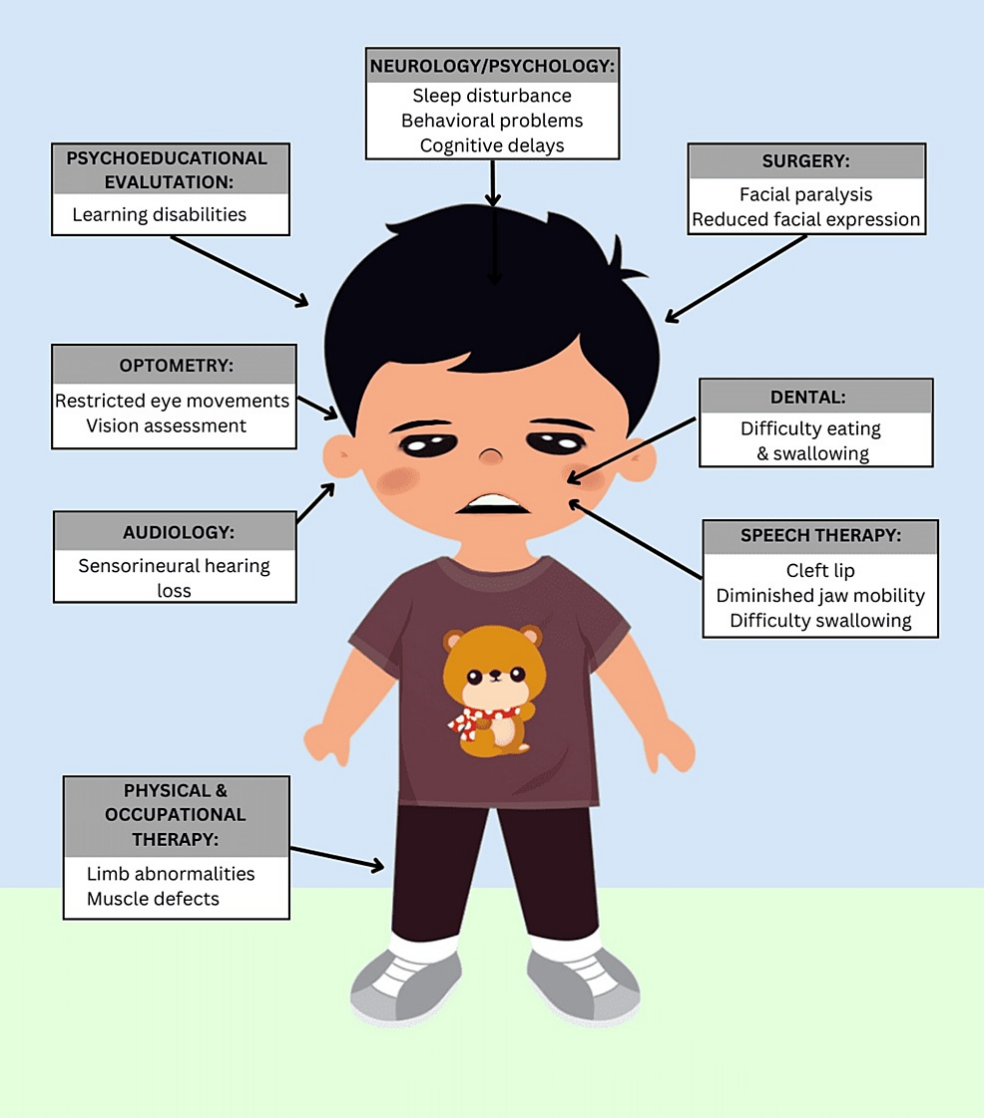
Manifestations of a Moebius Syndrome Patient Requiring Medical Referral The figure demonstrates the clinical symptoms experienced by this patient with Moebius syndrome and each respective necessary medical referral. The patient’s familial financial constraints and the overall limited resources available for uninsured patients prohibited access to the multitude of medical specialists needed until Medicaid was obtained.

Below in Figure 1 is a demonstration of the numerous specialists needed for our Moebius patient and why they were needed. Only the symptoms experienced by our patient are displayed in the figure.

In this case, while some nonprofit organizations were available to help manage our patient, the overall extent of care falls far below what is recommended for this child and others with similar needs. The current gap that exists in the treatment of low-income, uninsured, immigrant, and special needs children ultimately leads to a higher risk of regression and worsened long-term outcomes for this population.

There are no standard guidelines for the treatment of Moebius syndrome. However, early rehabilitation through physical, behavioral, and speech therapy can further improve the long-term physical and psychological prognosis of children with Moebius syndrome. Surgical interventions can help correct orofacial and limb deformities [9]. All of these were necessary treatments for our patient, however, his initial uninsured status made it extremely difficult to obtain them. Clinicians faced with uninsured patients with complex congenital diseases such as Moebius syndrome will need to seek out resources from the community such as local non-profit organizations, school systems, and free clinics until insurance can be obtained.

## Conclusions

Moebius syndrome is a rare congenital disorder characterized by non-progressive uni- or bilateral abducens (cranial nerve VI) and facial (cranial nerve VII) palsy. Other cranial nerves in addition to craniofacial and orthopedic anomalies may be involved leading to varied presentations. Treatment involves supportive care, possible early surgical intervention, and longitudinal, interdisciplinary care. This was a case of a 13-year-old male with Moebius syndrome seeking medical care hindered by multiple barriers including low income, initial lack of insurance, immigration status, and a language barrier. This patient required significant support from free community resources to improve long-term outcomes. These resources offered some support, however, overall access to sufficient care was limited. It was not until the patient received Simply Medicaid health insurance that he was able to receive the care he needed. Due to these limitations, physicians need to be well-versed in the community resources available to patients who lack insurance and are unable to afford medical services out-of-pocket until insurance or other financial support is obtained.
